# Evaluation of the Effects of Caesalpinia crista on Letrozole-Induced Models of Polycystic Ovarian Syndrome

**DOI:** 10.7759/cureus.34215

**Published:** 2023-01-25

**Authors:** Anagha Shende, Shirish Joshi, Paresh G Koli

**Affiliations:** 1 Pharmacology and Therapeutics, Seth Gordhandas Sunderdas (GS) Medical College and King Edward Memorial (KEM) Hospital, Mumbai, IND

**Keywords:** polycystic ovarian syndrome (pcos), ayurveda, menstrual disorders, animal study, caesalpinia crista

## Abstract

Background

Polycystic ovarian syndrome (PCOS) is the most common endocrine disorder in women of reproductive age, affecting reproductive, endocrine, and metabolic functions. This study was designed to validate the claims in Ayurveda regarding the efficacy of *Caesalpinia crista* (Latakaranj) to treat PCOS. Its seeds are uterine stimulants and ovulation inducers and improve menstrual cycle irregularities.

Objectives

The present study aimed to evaluate the effects of *Caesalpinia crista* on reproductive abnormalities, reproductive hormones, and glycemic changes in a letrozole-induced model of PCOS.

Material and methods

The study was performed in rats with six groups having six rats in each group. The control group was given the vehicle carboxymethylcellulose (CMC) for 21 days orally, followed by normal saline (0.9% NaCl) orally for 15 days. The inducing agent, letrozole, was given to the disease control group and the four treatment groups for 21 days, followed by a treatment period of 15 days with either clomiphene citrate (1.8 mg/kg) orally in the clomiphene group, low-dose (100 mg/kg) *Caesalpinia crista*, medium-dose (300 mg/kg) *Caesalpinia crista*, or high-dose (500 mg/kg) *Caesalpinia crista*. The variables assessed were daily vaginal smears to check for estrous cyclicity, body weight, blood glucose, serum testosterone (T), serum luteinizing hormone (LH), serum follicle-stimulating hormone (FSH), and the number of oocytes from each oviduct. Histopathology of ovaries was also done.

Result

There was no significant difference between the different groups for body weight and blood glucose. There was a significant difference between the regularity of the estrous cycle of the disease control group and the high-dose *Caesalpinia crista* (500 mg/kg) group (p<0.01). Hormonal levels of luteinizing hormone (LH) (p<0.05) and follicle-stimulating hormone (FSH) (p<0.05) were significantly raised in the high-dose *Caesalpinia crista* group, and that of testosterone was significantly decreased (p<0.05) in the high-dose *Caesalpinia crista* group compared to the disease control group. The number of ova was significantly high in the high-dose *Caesalpinia crista* group compared to the disease control group (p<0.05). Decreased number of atretic follicles was seen in the high-dose and medium-dose *Caesalpinia crista *group on histopathology, with an increased number of corpus lutea (p<0.05).

Conclusion

Treatment with *Caesalpinia crista* in high dose, i.e., 500 mg/kg, significantly improved the reproductive abnormalities (ovulation and menstrual irregularities) and histopathological changes associated with PCOS. It also restored reproductive hormone levels (testosterone, FSH, and LH), which are elevated in PCOS, and normalized the LH/FSH ratio, which is deranged in PCOS.

## Introduction

Polycystic ovarian syndrome (PCOS) is a heterogeneous and complicated condition that negatively affects a woman’s metabolic and reproductive health [1]. Its key characteristics include hyperandrogenism, which prevents follicular development, ovarian microcysts, anovulation, irregular menstrual cycles, and polycystic ovary shape. Infertility, menstruation irregularity or absence, indications of excess androgen, and obesity are some of its clinical symptoms [2].

Studies have found that the incidence of PCOS in Mumbai is 22.5%, and in the rural population, the incidence is 8.4% [3,4]. The commonly implicated causes for PCOS are genetics, obesity, sedentary lifestyle, stress, consumption of fast food and contraceptive pills, and intrauterine androgen exposure [5]. Firstly, menstrual irregularities, polycystic ovaries, and hirsutism are accountable for a rise in follicle-stimulating hormone (FSH) and luteinizing hormone (LH) levels, thus causing a reversal of the LH/FSH ratio. Secondly, insulin resistance-induced hyperinsulinemia, which indirectly raises androgen levels, is also involved [6-9].

There is not a perfect medical treatment for PCOS yet that completely corrects underlying hormonal imbalances and addresses all clinical symptoms [10,11]. The Endocrine Society recommends hormonal contraceptives to treat hirsutism and irregular menstrual cycles, metformin for metabolic and glycemic abnormalities, and clomiphene for ovulation induction. It also suggests that lifestyle modifications such as exercise and dietary changes can restore regular menstrual cycles [10]. The adverse effects, such as multiple pregnancies, ovarian enlargement, and ovarian hyperstimulation syndrome (OHSS), are related to hormonal contraceptives and clomiphene in young women and warrant a newer treatment with fewer adverse effects [12,13].

The estrous cycle is the reproductive cycle in rodents. It is similar to the human reproductive cycle, commonly called the menstrual cycle. The estrous cycle has four phases: proestrus, estrus, metestrus, and diestrus. The cycle length of the proestrus phase is 14 hours, estrus is 24-48 hours, metestrus is 6-8 hours, and diestrus is 48-72 hours. The reproductive period and estrous cycle of mice begins about the 26th day after birth with the opening of the vagina, which is about 10 days before vaginal cornification. In female rats, at approximately 30 days of age, there is a pulsatile release of luteinizing hormone (LH) followed by puberty. This period is the anestrus and occurs about 8-9 days before the first proestrus. Metestrus only occurs in the absence of conception.

In humans, there are three phases of the menstrual cycle: the menstrual, proliferative (follicular), and secretory (luteal) phases. The proestrus phase corresponds to the human follicular stage, which is associated with a rise in circulating estradiol concentrations and little surge in prolactin; this leads to a rise in LH and follicle-stimulating hormone (FSH) release. The peak in FSH concentration with an associated rapid decline in estradiol levels correlates to ovulation and the estrus phase. Metestrus and diestrus are homologous to human early and late secretory stages of the reproductive cycle, respectively, with high levels of progesterone [14].

Large, scandent, prickly *Caesalpinia crista* (*C. crista*) shrubs are often found in southern India and are frequently planted as hedge plants. Ayurveda literature states that *C. crista* can be used as a uterine stimulant and ovulation inducer and for menstrual cycle irregularities [15]. These properties, however, have not been studied before. Therefore, this study was undertaken and designed to validate the claims in Ayurveda regarding the efficacy of *C. crista* for PCOS treatment.

This study’s primary goal was to assess how *Caesalpinia crista *affected PCOS-related reproductive abnormalities, hormonal parameters, and glycemic changes.

## Materials and methods

Experimental animals

Prepubertal female Wistar rats (n=36) aged 4-6 weeks weighing 180-230 g were included in the experiment. In the Central Animal House (Seth Gordhandas Sunderdas (GS) Medical College and King Edward Memorial (KEM) Hospital, Mumbai, India), where controlled conditions were preserved with a temperature of 23°C±4°C and a humidity of 30%-70%, they were kept in standard laboratory conditions. Individual polypropylene cages (one per animal) with stainless steel top grills and amenities for delivering water and food were employed to keep the animals. In the cages, paddy husk served as bedding. Animals were provided with free access to UV-filtered water and pellet-based food (Chakan Oil Mills, Maharashtra, India). Twelve hourly light and dark cycles were maintained. The whole investigation was conducted under the standards established by the Committee for the Purpose of Control and Supervision of Experiments on Animals (CPCSEA), India. Before the research began, approval from the Institutional Animal Ethics Committee (AEC/04/2017) was acquired.

Study drugs and chemicals

The test drugs, the ethanolic extracts of *Caesalpinia crista* (*C. crista*), were used as study drugs. They were obtained as gift samples from Alarsin Pharmaceuticals (Mumbai, India) with a certificate of analysis. *Caesalpinia crista* was a brown-colored soft ethanolic extract with an extractive value of 11% w/w. In Ayurveda texts, doses are mentioned in terms of the crude powder of *Caesalpinia crista*. The doses for the ethanolic extract were selected from the published literature [16,17] on *C. crista* and after consultation with experts in Ayurvedic medications and research. Oral administration of 100, 300, and 500 mg/kg of ethanolic extract was selected.

The chemicals used in the study were letrozole (used as an inducing agent) at a dose of 1 mg/kg, which was obtained from Sun Pharma (Mumbai, India) as a gift sample, and clomiphene citrate, which was used as an active (positive) control to treat PCOS. The dose used was 1.8 mg/kg, and it was purchased from Sigma-Aldrich, India. The vehicles used for the drugs were 1% carboxymethylcellulose (CMC) as the suspending agent for letrozole, and normal saline was used as the vehicle for the active control of clomiphene citrate and the test drugs (*C. crista*).

Experimental phase

Six groups in the experimental phase comprised six female rats each (Table 1). The female Wistar rats were randomly allocated to six groups with random code generated in Microsoft Excel (Microsoft Corp., Redmond, WA, USA). The rats were given inducing agents for 21 days (day 1-21), drugs were given for 15 days (day 22-36), and finally, blood and histopathology tissue samples were taken on day 37.

**Table 1:** Experimental groups CMC: carboxymethylcellulose, *C. crista*: *Caesalpinia crista*

Groups (n=6/group)	Inducing agent (day 1-21)	Drugs given (day 22-36)
Vehicle control	1% CMC (2 mL) for 21 days	Normal saline (2 mL) for 15 days
Disease control	Letrozole (1 mg/kg) for 21 days	Normal saline (2 mL) for 15 days
Clomiphene	Letrozole (1 mg/kg) for 21 days	Clomiphene citrate (1.8 mg/kg) for 15 days
Low-dose *C. crista*	Letrozole (1 mg/kg) for 21 days	*C. crista* (100 mg/kg) for 15 days
Medium-dose *C. crista*	Letrozole (1 mg/kg) for 21 days	*C. crista* (300 mg/kg) for 15 days
High-dose *C. crista*	Letrozole (1 mg/kg) for 21 days	*C. crista* (500 mg/kg) for 15 days

The vehicle control group was orally given 2 mL of 1% carboxymethylcellulose (CMC) for 21 days. CMC was used as the suspending agent for the inducing agent letrozole. This was followed by 15 days of 2 mL of normal saline, which was the vehicle for the study drugs and active control. The disease control group was given letrozole in the dose of 1 mg/kg orally, suspended in 2 mL of 1% CMC for 21 days. Following induction with letrozole, the rats were given 2 mL of normal saline for 15 days. The natural course of the disease, PCOS, was seen in this group.

The positive control group (clomiphene group) was given letrozole in the dose of 1 mg/kg orally, suspended in 2 mL of 1% CMC for 21 days. After this, the rats were given clomiphene citrate in a dosage of 1.8 mg/kg orally dissolved in 2 mL of normal saline for 15 days, and this group served as a comparator for the treatment with the study drugs. Low, medium, and high dosages of the study drug were administered to the treatment groups. These groups will be given letrozole in the dose of 1 mg/kg orally, suspended in 2 mL of 1% CMC for 21 days as an inducing agent for PCOS. This was followed by the study drug for 15 days in 2 mL of normal saline. The *Caesalpinia crista* ethanolic extract was administered orally for 15 days at dosages of 100 mg/kg, 300 mg/kg, and 500 mg/kg in 2 mL of normal saline to the low-, medium-, and high-dose groups.

Variables assessed

Vaginal smears were taken daily to provide information on estrous cyclicity at various intervals during the study. The smears were taken by the swab smear technique at 9 am every day. Once each week, a mono-pan digital weighing scale was used to record the rats’ total body weight. The weight was recorded every week in the morning. Blood glucose levels were recorded every week. Blood (1 mL) was collected every week from the rats by the retro-orbital method. To stop glycolysis, the blood was drawn into a bulb carrying sodium fluoride (10 mg/mL blood). These blood samples were used to determine the blood glucose levels by Trinder’s method. Ovulation was checked by counting the number of oocytes from each oviduct. Serum LH, FSH, and testosterone (T) estimations were done using the enzyme-linked immunosorbent assay (ELISA) method on day 37. The ELISA kits were procured from KinesisDx, USA. Histopathology of the ovaries was done to check for the number and size of the ovarian cysts. The dissected ovaries stored in 10% buffered formalin were processed to prepare paraffin blocks, and the slides were prepared. They underwent eosin and hematoxylin staining. The cystic and atretic follicle number was counted from each specimen. We also counted and compared the numbers of corpus luteum and the primary and secondary follicles between the groups.

Statistical analysis

Findings were presented as mean ± standard deviation (SD). A p-value of <0.05 was considered statistically significant. The blood glucose within the group for the two groups was compared using repeated measures analysis of variance (ANOVA) during standardization. The variables body weight, blood glucose, vaginal smears, ovulation, serum testosterone, serum LH, and serum FSH were compared between the groups using one-way ANOVA with post hoc Tukey-Kramer multiple comparisons test. p<0.05 was considered significant. The statistical analysis software GraphPad InStat (Graphpad Software Inc., San Diego, CA, USA) version 3.06 was utilized.

## Results

All the groups’ average rat weights and blood glucose levels are shown in Table 2. At the end of the study, there was no statistically significant difference between the groups for both weight and blood glucose levels. There was no mortality or adverse effect observed in the rats.

**Table 2:** Average body weight at day 36 (n=36) Data represents mean±SD. p>0.05 using one-way ANOVA. SD: standard deviation, ANOVA: analysis of variance

Study group	Body weight (g)	Blood glucose (mg/dL)
Vehicle control	199.8±13.94	97.33±7.91
Disease control	203.5±7.09	95.83±12.50
Clomiphene	201.3±7.61	85.56±9.01
Low-dose *Caesalpinia crista*	206.1±18.61	97.3±7.91
Medium-dose *Caesalpinia crista*	202.5±6.05	99.36±8.29
High-dose *Caesalpinia crista*	208.6±7.87	87±6.61

The average number of days of each phase of the estrous cycle, i.e., proestrus, estrus, metestrus, and diestrus phase, were as given in Table 3. The number of days of the diestrus phase (similar to the late secretory phase in humans) increased statistically significantly in the disease control group compared to the vehicle control group. Moreover, there was a statistically significant difference between the disease control group and the vehicle control group in the estrus phase (similar to ovulation in humans) of the estrous cycle. The high-dosage *Caesalpinia crista* group experienced the diestrus phase for a similar number of days as the clomiphene group. Also, the number of days of the estrus phase was comparable to that of the clomiphene group.

**Table 3:** Number of days of estrous cycle phases (n=36) Data represents mean±SD. p>0.05 versus clomiphene group using one-way ANOVA. SD: standard deviation, ANOVA: analysis of variance

	Metestrus phase	Diestrus phase	Proestrus phase	Estrus phase
Vehicle control	7±1.67	14±1.41	4.2±2.80	8.8±0.41
Disease control	5.33±1.51	21.5±1.22	3.33±1.37	5.5±0.84
Clomiphene	5.83±1.47	18.83±1.60	4.16±1.72	6.83±0.75
Low-dose *Caesalpinia crista*	5±2.10	21.83±2.71	3.66±1.37	5.33±1.21
Medium-dose *Caesalpinia crista*	5.33±1.63	19.83±1.17	4±1.67	6.33±0.52
High-dose *Caesalpinia crista*	5.33±1.97	18.66±1.86	4.66±1.97	6.83±0.75

**Figure 1:**
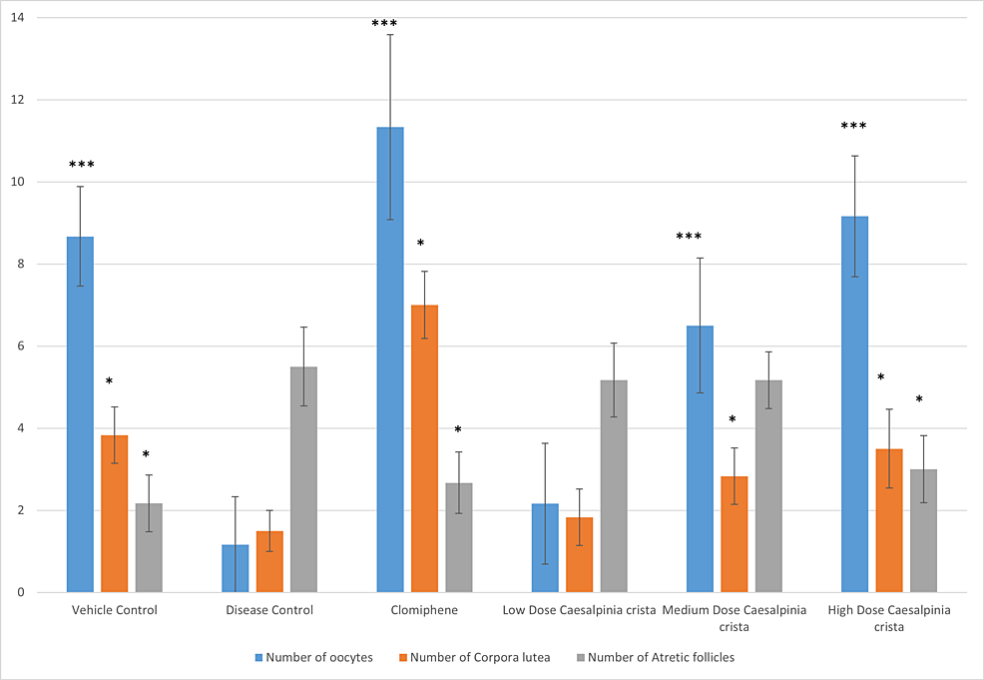
Number of oocytes, corpus luteum, and atretic follicles in ovaries (n=36) Data represents mean±SD. * is p<0.05, and *** is p<0.001 versus disease control group using one-way ANOVA with Tukey’s post hoc test. SD: standard deviation, ANOVA: analysis of variance

The average number of oocytes in all groups was as given in Figure 1. Compared to the disease control group, there was a statistically considerable rise in the number of oocytes in the clomiphene group, the vehicle control group, the medium, and the high-dose *C. crista* group. Comparable to the clomiphene group, the high-dose group had a similar number of oocytes.

The sections of ovaries from the vehicle control group showed healthy follicles with oocytes at different stages of development. Few follicles were seen in the early phases of development, and there was a rise in the atretic follicle number in the disease control and low-dose groups. These groups also showed subcapsular cysts with a thin coating of granulosa cells. The number of corpus lutea was decreased in the disease control group, thus revealing decreased ovulation. Compared to the disease control group, the number of corpus lutea was greater in the high- and medium-dose groups, as seen in Figure 1.

Serum testosterone levels in the disease control group were high, showing similar biochemical features of PCOS in humans. The total serum testosterone level in the active control group and the high-dose group decreased statistically significantly compared to the disease control group. The levels of serum testosterone in the medium- and high-dose groups were similar to those in the active control group, as seen in Table 4.

**Table 4:** Serum testosterone, LH, and FSH (n=36) Data represents mean±SD. *** is p<0.001 versus disease control group, and # is p<0.05 versus medium-dose group using one-way ANOVA with Tukey’s post hoc test. LH: luteinizing hormone, FSH: follicle-stimulating hormone, SD: standard deviation, ANOVA: analysis of variance

	Testosterone	LH	FSH
Vehicle control	86.33±8.98*	4.16±0.26*	8.54±0.37*
Disease control	211±8.39	2.83±0.17	1.98±0.26
Clomiphene	78.29±16.49*	3.91±0.31*	7.85±0.47*
Low-dose Caesalpinia crista	198.33±9.16	2.86±0.20	4.24±0.42
Medium-dose Caesalpinia crista	109±16.20	3.57±0.29*	7.07±0.22*
High-dose Caesalpinia crista	92.66±14.62*	4.02±0.28*^#^	7.39±0.43*

High levels of LH and FSH signify better follicle development and ovulation. Comparing the disease control group to the clomiphene group, medium-dose group, and high-dose group, there was a statistically considerable rise in the overall level of blood LH. The high-dose and medium-dose groups had serum LH levels equivalent to those of the active control group. The high-dose group’s blood LH level is equivalent to that of the medium-dose group.

Compared to the disease control group, the total level of serum FSH was elevated in the clomiphene group, medium-dose group, and high-dose group. The high-dose group’s serum FSH level was equivalent to the active control group.

## Discussion

PCOS is a distressing condition for women, sometimes complicated for clinicians, and a complex scientific problem for investigators. PCOS is a heterogeneous disorder that can be identified by a combination of symptoms and indications of androgen excess (hyperandrogenism and/or hirsutism) and ovarian deregulation (polycystic ovarian morphology (PCOM) and/or oligo-ovulation), provided that other indications, including hyperprolactinemia and nonclassical congenital adrenal hyperplasia, have been ruled out [18].

According to recent, more comprehensive definitions, the incidence of PCOS in premenopausal females varies from 20%-6%, potentially making this syndrome the most common metabolic and endocrine illness in females of reproductive age [6]. The therapy of PCOS is now focused on symptoms rather than a particular etiologic pathway, since the underlying pathophysiology of the condition is not completely known. A healthier lifestyle and losing weight have been shown to lessen the effects of androgens, improve ovulation, and increase insulin sensitivity. Metformin lowers testosterone levels; however, it has been shown to have minimal effect on fertility and little impact on how insulin works. According to earlier reports, thiazolidinedione enhances insulin action without changing ovarian function. Increasing fertility with the potential for multiple pregnancies, clomiphene, and aromatase inhibitors do not affect the condition’s other metabolic or psychosocial signs. There is evidence that surgery enhances fertility and transiently alters ovarian function. In the cycle being treated, ovarian gonadotropin stimulation may enhance ovarian function in PCOS, but the treatment carries an elevated risk for multiple births and ovarian hyperstimulation syndrome (OHSS). Advanced reproductive treatments carry the potentially fatal danger of OHSS in PCOS patients.

Hirsutism may be mitigated, but not entirely, by antiandrogens. However, the pharmaceutical sector and international and local health authorities have not shown the same interest in PCOS as the scientific community. Stein and Leventhal initially identified PCOS in 1935 as a condition defined by hirsutism, a terminal hair growth condition in women with a male pattern, amenorrhea, obesity, chronic anovulation, infertility, and enlarged cystic ovaries. However, the International Classification of Diseases, 10th edition, did not recognize “E28.2 polycystic ovarian syndrome” (also called Stein-Leventhal or sclerocystic ovary syndrome) as a disease of ovarian dysfunction until 1990. Because neither the Food and Drug Administration (FDA) nor the European Medicines Agency has ever authorized a medication, particularly for PCOS treatment, most medicines that are taken for PCOS treatment, from oral contraceptives to insulin sensitizers, aromatase, or antiandrogen inhibitors, are used off-label. Clinical studies that have recently been registered serve as an example of the pharmaceutical sector’s minimal interest in PCOS: at the end of August 2017, there were 4,632 research addressing diabetic mellitus, compared to 28 commercial studies on PCOS, despite both conditions having comparable global incidence. Therefore, from the aspect of pharmaceutical therapy, PCOS may now be regarded as the most common orphan condition affecting adolescent and adult females.

The most likely reason for the lack of interest by health officials and the pharmaceutical sector is that PCOS continues to be one of the medical conditions that patients, doctors, and even scientists do not fully comprehend. As a result, there has not been a lot of funding for research and development in this field because of the general lack of understanding of the condition and its long-term effects on the health of persons and their families. The inadequacy of PCOS’s naming, its heterogeneous nature, the perennially divisive question of its definition, and several unanswered questions about its origin and pathophysiology are some causes of this misinformation [6]. Also, there are no approved drugs to treat PCOS by the FDA owing to the lack of clinical trials and the side effect profile associated with the use of drugs used to treat the symptoms related to PCOS. As a result, many patients have turned to alternative systems of medicine such as Ayurveda and homeopathy. Given this, we conducted a study that assessed the effects of the seeds of the *Caesalpinia crista* plant on PCOS.

Ayurveda physicians routinely use *Caesalpinia crista* in patients presenting with symptoms of PCOS and in cases of infertility. It is also mentioned as a uterine stimulant that improves menstrual discharge in oligomenorrhea and reduces the pain in the lower abdominal region associated with dysmenorrhea. Thus, we wanted to validate the claims of Ayurveda in this preclinical animal study using a letrozole-induced model of PCOS, as it simulates the condition of human PCOS the most. Ethanolic extract of the plant *Caesalpinia crista* was used as the test drug. The seeds contain a fatty oil containing sitosterol, a hydrocarbon, and two phytosterols. The seeds include a substantial proportion of thick, pale-yellow oil, which has an unpleasant smell. It has a saponification value of 292.8 and an iodine value of 96.1. The cotyledons of the seed also contain a nonalkaloidal bitter substance called natin that is soluble in alcohol and chloroform. The seeds contain a hard outer shell that makes up 58% of the seed, and the remaining 42% is the kernel. The bonduc in a white powder extracted from the kernels and responsible for the physical characteristics of the seed is a bitter nonalkaloidal principal. It was discovered that it was soluble in oils but insoluble in water. *Caesalpinia crista* has been the subject of previous chemical investigations that led to the isolation of flavonoids and cassane furanoditerpenes. Numerous diterpenoids, including caesalpinolide-C, caesalpinolide-D, caesalpinolide-E, and cassane furanoditerpene, are present in the plant *Caesalpinia crista* [19-21]. Reddy et al. (2018) published the *Caesalpinia crista* seed extract LD50 result; therefore, dosage levels of 100 mg/kg, 300 mg/kg, and 500 mg/kg were chosen as the low, medium, and high dosage for the treatment [22]. There is growing evidence that shows that irregular programming of developing systems during prenatal life may cause adult dysfunctions. There is a strong indication that an early androgen excess may cause the adult phenotype of PCOS. This is confirmed by the fact that the PCOS phenotype is also linked to diseases such as classical 21-hydroxylase insufficiency, which occurs when the fetus was exposed to excessive levels of sex steroids before birth. The term “androgenized models” refers to most animal models that exhibit the PCOS phenotype and entail prenatal testosterone (T) treatment. Since T may aromatize to estrogen and exhibit its effects through estrogenic programming, this nomenclature is often misleading. Other models include prenatal exposure to inhibitors, estrogenic compounds, or non-aromatizable androgens such as dihydrotestosterone (DHT).

The most in-depth research has been done on rats, mice, rhesus monkeys, and sheep among the animal models used to understand the PCOS etiology. These models all have various advantages. It is undeniable that rhesus monkeys are the best genetically. Still, their general use in research is limited by cost prohibitiveness and their long developmental lines (menarche occurs at around 2.5 years of age, and reproductive competence between 2.5 and 3.5 years of age). Rodents are now the most popular animal used to investigate PCOS because of their advantages in terms of short lifespan, size, high reproduction index, and variety of genetic strains [23,24]. The essential enzyme, aromatase, converts T and androstenedione to estrone and estradiol. It is extensively expressed in human tissues, including the placenta, testis, and ovary. One of the pathophysiologic theories explaining the onset of PCOS is decreased aromatase activity in the ovary. Letrozole, a nonsteroidal aromatase inhibitor, increases the synthesis of T and decreases estradiol production by reducing the conversion of androgens to estrogens in the ovary. In rats receiving letrozole treatment, excess T in the ovaries is probably the direct cause of polycystic ovaries. By weakening the negative effect on LH production in the pituitary, the loss of estrogen leads to higher levels of LH, which further stimulate theca cells to release T. Letrozole is typically given orally to six-week-old female rats (puberty) at dosages of 0.1, 0.5, and 1 mg/kg daily for 21 days, after which they become acyclic and exhibit biochemical and histological characteristics of human PCOS. The letrozole model is intended to investigate classic PCOS caused by aromatase insufficiency and may be a helpful cotreatment with other therapies that cause cardiometabolic abnormalities [25].

Clomiphene citrate was chosen as the positive control for the present study. Luteinizing hormone (LH) and follicle-stimulating hormone (FSH) levels are raised by the weak estrogen-like hormone clomiphene, which works on the brain, pituitary gland, and ovary (LH is crucial for the ovulation process). A preovulatory gonadotropin surge and subsequent follicular rupture result from a sequence of endocrine processes triggered by clomiphene citrate. An increase in pituitary gonadotropin release is the initial endocrine reaction after a course of clomiphene medication. This starts the processes of steroidogenesis and folliculogenesis, which cause the ovarian follicle to expand and the estradiol level in the blood to rise. Plasma levels of estradiol and progesterone increase and decrease after ovulation, just as they would during a regular ovulatory cycle. The probability of developing an ovarian follicle, which may subsequently cause ovulation, increases with a rise in FSH levels. About 80% of women who take clomiphene for irregular ovulation will ovulate, and 30%-40% of all women on clomiphene become pregnant [26]. The model was standardized in the standardization phase. Administration of 1 mg/kg letrozole for 21 days showed an irregularity of the estrous cycle deduced by the increase in the day count of the diestrus phase and a decrease in the estrus phase number of the cycle in the disease control group. The disease control group’s lower ovulation was seen in the histopathology through a decline in the number of corpora lutea.

Histopathology revealed an increase in atretic follicles in the disease control group. The body weight and blood glucose did not show any significant changes. The findings of the standardization phase were consistent with those of the study performed by Dăneasă et al. [27] and Kafali et al. [28]. As a result, the letrozole-induced PCOS model was standardized.

The body weight and total blood glucose did not show any remarkable changes in the test group compared to the vehicle control and clomiphene group. The letrozole-induced model of PCOS did not mimic the glycemic changes that occur in PCOS. However, since the test drug has antidiabetic properties, we wanted to analyze the impact of the test drug *C. crista* on the total body weight and blood glucose. The normal estrous cycle in a rat is, on average, four days, which could range from three days to five days. In PCOS, the day count of the diestrus phase increases, whereas the day count of the estrus phase decreases. With the administration of clomiphene, the estrous cycle was normalized. Similar changes were seen with a high dose of the test drug *C. crista* (500 mg/kg). This is to the claims in Ayurveda texts of the drug being used for menstrual irregularities [29,30]. *Caesalpinia crista* showed a rise in the oocyte number after dissection with medium dose and high dose, which was statistically significant compared to the control group. Comparable to the clomiphene group, the high-dosage group had a rise in the oocyte count. The increase in the number of ova must be due to the property of the test drug to act on the Bija property in females, as stated in Ayurveda texts. On histopathology of the ovary, the number of corpus lutea in the medium- and high-dose group was statistically considerably raised compared to the disease control. This thus reflected the increased ovulation in these groups.

Additionally, there was a statistically significant decline in the number of atretic follicles in the high-dose *C. crista* group compared to the disease control group. It was also comparable to that in the clomiphene citrate group. Ideally, histopathology of the ovary requires serial sections of the whole ovary. This is required to study the cysts’ size and appropriately count the number of cysts as well as the corpus lutea. The same corpus lutea or the cysts can reappear in the next section and be counted twice if serial sections are not taken.

Similarly, some of the cysts or corpus lutea can be missed. However, in our study, it was not feasible to do serial sections; therefore, the sections were taken as explained in the materials and methodology section. The elevated testosterone levels in the disease control group prove that PCOS causes a rise in testosterone levels. Compared to the disease control group, the serum testosterone level in the *C. crista* group receiving the medium and high doses reduced statistically substantially. Also, the level of testosterone in the medium- and high-dose *C. crista* group was comparable to that in the clomiphene citrate group. This drop in serum testosterone helps reduce the number of atretic follicles and normalize the estrous cycle.

The level of serum LH and FSH increases in PCOS, so it causes a reversal of the LH/FSH ratio. The normal LH/FSH ratio is 1:2, which was also seen in the vehicle control group. This ratio is reversed in PCOS to 2:1-3:1. The disease control group’s LH/FSH ratio was 2:1.4. This ratio was normalized in the clomiphene group (1:2) as well as the medium-dose group (1:2). The estimation of the level of serum estrogen could also have been done to check if the plant had estrogenic properties and correlated with the histopathological results and other hormonal parameters. However, these investigations could not be carried out because of limited resources.

Nevertheless, the findings of this report clearly show that *Caesalpinia crista* exerts a positive effect against letrozole-induced PCOS in female Wistar rats.

## Conclusions

Treatment with *Caesalpinia crista* in high dose, i.e., 500 mg/kg, significantly improved the reproductive abnormalities (ovulation and menstrual irregularities) and histopathological changes associated with PCOS. It also restored the reproductive hormone levels (testosterone, FSH, and LH), which are elevated in PCOS, and normalized the LH/FSH ratio, which is deranged in PCOS. Thus, the findings of this report prove that *Caesalpinia crista* in high dose (500 mg/kg) exerts a positive effect against letrozole-induced PCOS in female Wistar rats. *Caesalpinia crista* needs to be evaluated in other models of PCOS to check for its effect on the metabolic abnormalities of PCOS, and additional research is needed to delineate its mechanism of action. *Caesalpinia crista* could be a potential drug for treating PCOS for clinical use in the future.
